# The Assessment of Natural Biomineral Leonardite on Growth and Pigmentation of Goldfish, *Carassius auratus*

**DOI:** 10.3390/life15010074

**Published:** 2025-01-10

**Authors:** Dilek Şahin, Meryem Öz, Ünal Öz

**Affiliations:** 1 Vocational School, Sinop University, 57000 Sinop, Turkey; 2 Fisheries Faculty, Sinop University, 57000 Sinop, Turkey; meryemoz@sinop.edu.tr (M.Ö.); unaloz@sinop.edu.tr (Ü.Ö.)

**Keywords:** ornamental fish, leonardite, pigmentation, feed additive, water conditioners

## Abstract

In this study, the impact of leonardite as a feed additive in goldfish diets was examined, focusing on its potential to positively influence feed and water parameters, which are critical for achieving sustainable ornamental fish farming. In this study, goldfish were fed diets containing four different levels of leonardite (0%, 2%, 6%, 10%). The experiment was conducted in 12 tanks with 3 replicates per treatment and lasted for 3 months. The fish, which were provided from the Research, Production and Training Institute, with an average live weight of 0.947 ± 0.03 g and an average length of 1.12 ± 0.02 cm, were used. After 90 days, it was observed that the specific growth rates increased in correlation with the amount of leonardite in the diet, with a statistically significant difference identified (*p* < 0.05). In this study, which investigated the coloration of fish with leonardite added to the feed for the first time, it was determined that the color of the fish approached from yellow to orange as the amount of leonardite added to the feed increased (*p* < 0.05). Given the positive effects observed on pigmentation and growth in this study, it is recommended that further detailed investigations be conducted on leonardite, a natural, harmless, and cost-effective additive, using different fish species.

## 1. Introduction

The aquaculture sector, along with ornamental fish farming, which is a significant component of this industry, has shown continuous development to meet increasing demand. Ref. [1] reported that recent studies estimate the annual trade volume of the aquarium fish industry to be between 15 and 30 billion USD. Most marine ornamental fish are collected from the wild, with only 1–10% of the species in the trade estimated to be captive-bred, in contrast to freshwater aquarium species, for which 95% are currently bred in captivity [2,3]. Within aquaculture, ornamental fish farming has become a highly prominent sector due to the diverse and vibrant colors, shapes, and behaviors of these fish. The ornamental fish industry contributes to conservation efforts by promoting the breeding and preservation of traditional aquarium fish species (e.g., goldfish, livebearers, cichlids, etc.) as well as endangered or threatened species. Over time, technological advances and their application in this sector have increased significantly. Not only have breeding techniques improved, but other areas such as disease prevention, nutrition, water quality management, and logistics have also improved, all of which have increased the production of live specimens and facilitated trade [4,5,6,7]. Ornamental fish farming with significant trade began over 1000 years ago in China with the domestication of the freshwater goldfish. Goldfish, a variety of *Carassius auratus*, is the most preferred species due to its ease of breeding, long lifespan, and variety of colors. Goldfish are omnivorous in their feeding habits. *C. auratus* Linnaeus, 1758, commonly known as goldfish, is particularly popular in aquarium fish markets [5,6,7,8].

Approximately 90% of freshwater ornamental fish production is carried out through aquaculture [3]. In aquaculture practices, nutrition and the regulation of water parameters are two critical factors influencing the success of production [9]. These two factors, which are interdependent, are managed using natural materials such as feed additives and water conditioning agents. Among these materials, naturally derived products like zeolite, bentonite, and leonardite, which contain minerals, clays, or humic substances, are utilized in various fields, and scientific research continues to explore new applications for them [10,11]. The properties of such natural materials are influenced by numerous factors, highlighting the need for research conducted under conditions specific to their intended applications [12]. It is essential to comprehensively determine the potential uses of these natural materials as both feed additives and water conditioners in aquaculture.

Feed additives are substances added in trace amounts to a diet or feed component to enhance or preserve its properties. In aquaculture, feed additives are incorporated into diets to meet the biological requirements of fish, such as growth, reproduction, and pigmentation [8,13]. Additionally, feed additives can play a significant role in regulating the immune system and reducing the adverse effects of stress in fish [13,14]. According to [15], the literature reviewed shows that the application of functional feed additives in aquaculture reduces stress, facilitates digestion, promotes growth, improves water quality, increases survival rates of aquatic animals exposed to infections, reduces parasite infestation and reduces the environmental impact of aquaculture. These feed additives, which provide a multitude of benefits, provide significant advantages to farmers by increasing profitability, reducing dependence on antibiotics and minimizing the costs associated with their purchase and the adverse effects associated with their use [15]. The numerous benefits derived from functional feed additives position them as ‘superfoods’ in aquaculture. Although the use of functional feed additives represents a significant breakthrough for the sector, further research is required to determine the optimum combination and dosage of these additives that will provide greater benefits than those currently available [15].

Humic substances are naturally found in water and soil and are considered part of the fish’s natural habitat due to their aquatic origin. Because of their unique properties, humic substances can be used as alternative feed additives and water conditioning materials in aquaculture [16,17,18]. Globally, there are various leonardite deposits with chemical compositions and humic acid contents [19]. Leonardite is a natural product composed primarily of humic acids derived from the decomposition of organic matter, and it is commonly used for soil fertilization. Furthermore, humic substances, as natural organic compounds, are found in aquatic systems and are compatible with various aquatic organisms. Adding humic substances to rearing water or fish feed can regulate bacterial communities associated with fish, thereby reducing the presence of harmful pathogens [5,13,16].

Humic acid generally exhibits many positive effects on poultry production, including feed performance, disease resistance, immune system function and stress management. The use of humic acid as a part of food supplements has been the subject of research in poultry. However, studies conducted on fish have mostly focused on humic acid extracts [20,21,22], with relatively few investigating the direct use of leonardite as a feed additive [11,13,23,24]. To meet the increasing demand for ornamental fish, the introduction of innovative and sustainable farming practices could lead to improvements in the aquaculture sector while also contributing to the conservation of natural resources in many developing countries [25].

Studies have highlighted the need for detailed investigations into the effects of feed additives (e.g., probiotics, prebiotics, humic acid substances/leonardite, etc.) on growth, reproductive performance and pigmentation in ornamental fish farming [7,11]. In this study, the impact of leonardite as a feed additive in goldfish diets was examined, focusing on its potential to positively influence feed and water parameters, which are critical for achieving sustainable ornamental fish farming.

## 2. Materials and Methods

### 2.1. Experimental Design

In the study, goldfish were fed diets containing four different levels of leonardite (0% (control), 2%, 6%, 10%) [10]. The experiment was conducted in 12 tanks with 3 replicates per treatment and lasted for 3 months. The control diet used was a commercial aquarium fish feed containing 48.5% protein, 6.7% fat, 1.9% cellulose and 10.4% ash. At the start of the research, the leonardite was thoroughly washed with water to remove turbidity and then dried at 105 °C [26]. The dried leonardite was sieved to obtain a powdered form used in this study. Commercial aquarium feed was moistened with warm water, powdered leonardite was added in the specified proportions (2%, 6%, and 10%) and the mixture was mixed homogeneously. The feeds that have absorbed the powdered leonardite were then dried at 60 °C.

The fish were provided from the Mediterranean Fisheries Research, Production and Training Institute, Antalya. The fish, with an average weight of 0.947 ± 0.03 g and an average length of 1.12 ± 0.02 cm, were randomly selected from a stock tank containing 250 fish and placed into each of the 30 L tanks, with 14 fish per tank, ensuring no statistical differences between groups. In studies involving fish in aquaculture, feeding is typically carried out until satiation or according to a percentage of body weight. In juvenile fish rearing, the satiation method is preferred to avoid stressing the juvenile fish during weighing procedures [27,28,29]. The fish were fed twice daily (09:00, 15:00) with four different experimental diets until satiation. It was determined that the fish had reached satiety through observation (until they ceased feeding). The amount of food consumed by the fish during the trial was subsequently recorded. Waste materials (uneaten feed and fish feces) in the experimental tanks were siphoned off weekly. After siphoning, the amount of water lost was replenished with water of the same characteristics.

The average water parameters used in the experimental tanks were set as follows: water temperature at 24 ± 1 °C, dissolved oxygen above 5 mg L^−1^, pH between 7.5 and 8.0 and ammonium levels below 1 mg L^−1^ [30]. The photoperiod was set to a natural light cycle (14 h of light: 10 h of darkness). Water temperature, pH level, dissolved oxygen, and NH_4_ concentration were measured using a YSI Professional Plus device. The color parameters of the fish were measured using a Konica Minolta CR-400 color measurement device.

**Figure 1:**
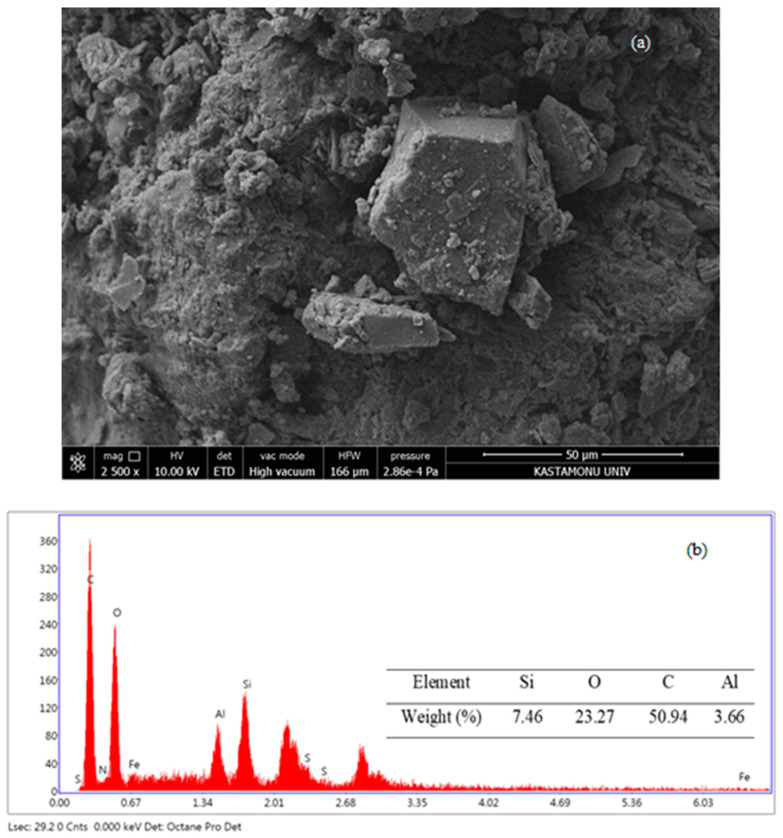
SEM images (**a**) and EDS analysis (**b**) of leonardite.

Leonardite used in this study were provided by Kütahya Kimya (Kütahya, Türkiye). Leonardite was characterized by SEM/XRF/BET (Table 1, Figure 1). These analyses were performed by the Central Research Laboratory of Kastamonu University. pH values were calculated according to [31]. The Brunauer–Emmett–Teller (BET) analysis and X-ray fluorescence (XRF) analyses of leonardite used in this study were carried out at Kastamonu University Central Research Laboratory using Quantachrome brand Nova Touch LX4 and Spectro brand Xepos II model devices, respectively. Scanning electron microscope (SEM-EDS) analyses for the natural adsorbents were performed using the FEI brand Quanta FEG 250 model instrument at the Kastamonu University Central Research Laboratory and the JEOL brand JSM 7001 F model device at the Ondokuz Mayıs University Karadeniz Advanced Technology Research and Application Center was used for the conditioned adsorbents.

### 2.2. Assessment of the Results

The weights of the fish were measured at the beginning and end of the trial using a KERN brand scale with accuracy of 0.001 g. The growth, survival rate and coloring parameters were calculated using the following equations [32];

Weight gain (g) = Final live weight (g) − Initial live weight (g)(1)

(2) $$Specific\;growth\;rate\;(\%)=\frac{lnWf-\ln Wi}{t-ti}\times 100$$

In the equation, Wf is the final weight (g), Wi is the initial weight (g) and (t − ti) is the length of the experiment (day).

(3) $$Feed\;conversion\;ratio=\frac{Total\;feed\;quantity\;consumed\;throughout\;the\;experiment\;(g)}{Total\;live\;weight\;gain\;(g)}$$

(4) $$Survival\;rate(\%)=\frac{Final\;number\;of\;fish}{Inital\;number\;of\;fish}\times 100$$

Color parameters were determined using the physical color (instrumental) identification method. Color measurements were made at the end of the trial and at the beginning of the trial under the same conditions (same time interval, same measurement location, same measurement distance, etc.). The same region of all fish was used for color measurement. A colorimeter (Konica Minolta CR 400) was used to measure the skin L*, a* and b* values from around the dorsal section. The C* (chroma) and Hab° (Hue angle) values were calculated using the a* and b* values. Color parameters determined were L*: (+) lightness, (−) darkness, a*: (+) redness, (−) greenness, b*: (+) yellowness, (−) blueness [33,34].

Chroma (Cab*) indicates the saturation, density or brightness of a color and calculated using the following equation;

Cab* = (a*^2^ + b*^2^)^½^ (5)

Hue indicates the relationships among yellowness, greenness and blueness of skin color. If a* > 0, then Hab° = tan^−1^ (b*/a*) is used, or if a* < 0, then Hab° = 180 + tan^−1^ (b*/a*)is used to calculate the hue angle [35]. The hue indicates a red color tone at 0°, a yellow color tone at 90°, a green color tone at 180° and a blue color tone at 270° [33,36,37].

### 2.3. Statistical Analyses

The data obtained in the research were statistically analyzed with the Minitab Package Program for Windows (version 17). The study initial parameters were compared with analysis of variance, and it was determined that the differences between groups were statistically insignificant (*p*  >  0.05). The normality of the data was confirmed by the Anderson–Darling, Ryan–Joiner (similar to Shapiro–Wilk) and Kolmogorov–Smirnov tests. When the variance analysis prerequisites were met, the data were subjected to parametric tests (ANOVA). In contrast, non-parametric (Kruskal–Wallis) tests were employed when the prerequisites were not met. The results were presented as mean ± standard error (SE), and a 0.05 margin of error was chosen in this study [38].

### 2.4. Ethics and Legal Aspects

This study protocol was approved by the Ethics Committee of Sinop University, and the experiments were conducted in accordance with the ethical guidelines and regulations set forth by Sinop University, as well as the international principles for the use and care of laboratory animals.

## 3. Results

### 3.1. Chemical Characterization of Leonardite

The characteristics of leonardite used in this study are given in Table 1. Leonardite was characterized by SEM/XRF/BET (Figure 1).

The chemical composition of leonardite revealed a high carbon (C) content, along with the presence of elements such as silicon (Si), aluminum (Al), calcium (Ca), sodium (Na) and phosphorus (P) (Table 1, Figure 1).

### 3.2. Water Parameters

Mean temperature, pH, dissolved oxygen and NH_4_ values of control (0%), 2%, 6% and 10% groups at the end of the experiments are shown in Table 2. The differences in the values of parameters recorded at the beginning and end of the experiment were not significantly different (*p* > 0.05).

### 3.3. Growth Parameters

In this study, which examined growth and pigmentation, leonardite was added to the diets at proportions of 0%, 2%, 6% and 10%. After 90 days, it was observed that the specific growth rates increased in correlation with the amount of leonardite in the diet, with a statistically significant difference identified (*p* < 0.05). When the feed conversion ratios were analyzed, the most optimal group was determined to be the group with 10% leonardite (0.681 ± 0.006), followed by the group with 6% leonardite (0.975 ± 0.032). Additionally, the highest survival rate was observed in the group containing 6% leonardite, with a rate of 90.48 ± 2.38% (Table 3).

At the end of this study, growth in length across all experimental groups was assessed. The growth values for the groups containing 0%, 2%, 6% and 10% leonardite were determined as 2.588 ± 0.093 cm, 2.72 ± 0.078 cm, 3.065 ± 0.094 cm and 3.203 ± 0.084 cm, respectively. Statistical analysis indicated that the 0% and 2% groups differed significantly from the 6% and 10% groups (*p* < 0.05).

### 3.4. Color Parameters

In this study, unlike previous research, it was determined that the pigmentation rate in fish fed with leonardite-supplemented diets was highest in the groups containing 6% and 10% leonardite. Skin color parameters of the experimental groups were measured at the beginning and end of the experiments. The L*, a*, b*, Hue (Hab°) and Chroma (C*) values are presented in Table 4.

In this study, which investigated the coloration of fish with leonardite added to the feed for the first time, it was determined that the color of the fish approached from yellow to orange as the amount of leonardite added to the feed increased, with H_ab_° values of 85.36 ± 4.68 in the groups fed with 6% leonardite-added feed and 94.56 ± 7.06 in the groups fed with 10% leonardite added feed (*p* < 0.05).

## 4. Discussion

Aquaculture feeds contain a wide variety of ingredients that meet the nutritional requirements of fish for growth, reproduction and immune function. To support feed intake, digestion, absorption and nutrient delivery to cells, an increasing number of non-nutritive feed additives are being incorporated into fish diets. These feed additives are utilized to enhance the physical and chemical properties of the feed, thereby contributing to the cultivation of aquatic organisms [39].

Leonardite, a primary source of humic substances, has the potential to serve as a feed supplement [11,40]. The use of humic acids (HAs) in aquarium fish has been a topic of research. Studies have found that humic acid is a potential feed and water additive with positive effects on overall fish performance, including growth, feed conversion, lysozyme and gut enzyme activity, health status, antioxidant capacity and stress parameters [41,42]. However, further investigation is required to fully understand its impacts on fish health and water quality parameters [43].

Humic acid also positively influences the immune system due to its beneficial properties, such as antibacterial, antiviral and anti-inflammatory effects [44]. While several studies have reported its positive effects on growth and immunity, other research has highlighted adverse impacts of humic acid supplementation on tissues such as gills and kidneys [45,46]. In another study on the addition of leonardite to feed, no significant differences in growth parameters were observed, emphasizing the importance of dosage optimization [13]. Ref. [47] reported that humic acid, when used at appropriate levels, can be a better alternative to chemical applications, improving growth performance, reducing mortality, enhancing immunity and mitigating the effects of stress factors. Leonardite, a source of humic acid, has been the subject of research on various topics, ranging from regulating water parameters to enhancing growth parameters in aquaculture, due to its natural availability and richness in humic substances [11,48].

Given this context, research into the direct use of leonardite, known as a natural source of humic acids, in fish feed or aquaculture water is of significant importance. In the present study, leonardite, in its natural form, was incorporated into fish feed, and its effects were evaluated.

Previous studies have shown that humic acid positively influences fish aquaculture. However, research on leonardite, a primary source of humic acids, in fish farming remains limited. For instance, [23] supplemented carp feed with 2% humic acid and 2% leonardite. Survival rates were reported as 71% in the humic acid group, 72% in the control group and 79% in the leonardite group. Similarly, [13] investigated the effects of leonardite supplementation at 0%, 0.5%, 1% and 3% in trout diets. After a 56-day trial, they found that fish weights increased compared to the baseline; however, no statistically significant differences in growth or survival rates were observed among the experimental groups.

In male *Betta splendens*, [42] reported that diets supplemented with humic acid had no significant effect on growth performance or feed conversion (*p*> 0.05). Nonetheless, fish fed with 1% humic acid exhibited numerically higher body weights compared to other groups. According to [11], humic substances are utilized as alternative feed additives in animal nutrition due to their natural growth-enhancing properties. Humic acid was found to increase villus length and improve growth performance as a result of enhanced nutrient absorption. In the study conducted by [11], it was determined that there was a correlation between growth and survival parameters and the increasing amount of leonardite added to the feed. In a related study conducted under similar conditions, [11] observed that a diet containing 5 g kg^−1^ leonardite had no adverse effects on survival rates and led to improved weight gain in goldfish.

Maintaining water parameters within suitable limits is essential for the success of aquaculture. Sudden changes in water parameters, especially pH, oxygen and temperature, cannot only cause stress in fish but also lead to mortality. Furthermore, these parameters can influence or be influenced by one another. Since humic substances are a natural component of the fish’s habitat, their defense mechanisms do not perceive these foreign substances as threatening, allowing them to continue their normal activities without any adverse reaction [47]. As a result of this study, when water parameters were examined, it was determined that leonardite had a positive effect on growth and survival parameters in goldfish.

This study, unlike previous research, identified that fish fed with leonardite-supplemented diets showed the highest pigmentation rates in groups containing 6% and 10% leonardite (Table 4). Skin pigmentation is one of the most critical quality criteria determining the market value of ornamental fish [49], and numerous studies have focused on the coloration of aquarium fish [50,51]. Fish skin pigmentation is regulated by both external (biotic and abiotic) and internal (genetic, cellular, neural and hormonal) factors [52]. In this study, feeding protocols, one of the external factors influencing pigmentation, were investigated. In this regard, this study represents the first to evaluate the effects of leonardite supplementation on skin pigmentation in ornamental fish diets. The research most closely related to this study was conducted by [24], who examined the effects of humic acid supplementation on the muscle pigmentation of trout. Their study found no statistically significant differences in L, a and b values among the groups regarding trout muscle coloration. In contrast, the current study demonstrated that supplementation with 6% and 10% leonardite positively influenced red pigmentation, with statistically significant differences compared to other groups.

Previous studies [51,53] reported that water quality plays an important role in enhancing the color quality or brightness of fish. In their studies, they found that maintaining water quality parameters, which are external factors influencing the body coloration of koi fish, within the ideal range has a positive effect. In the present study, it was determined that the addition of leonardite to the feed did not negatively affect the optimal water parameters required for goldfish, which are cyprinids. Considering the positive results obtained in this study, which is the first to investigate the effect of leonardite addition on color, it is suggested that future research should explore this effect in different fish species.

## 5. Conclusions

In ornamental fish aquaculture, pigmentation and growth parameters are the two most critical factors. The faster a fish reaches marketable size and coloration, the greater the commercial success. The findings from this study can be summarized as follows: As the amount of leonardite added to the feed increased, there was a corresponding positive effect on fish weight and length growth. The skin pigmentation of fish, a key driver of the ornamental fish industry, increased proportionally with the amount of leonardite included in the diet. This research is the first study to investigate the impact of leonardite as a feed additive on pigmentation.

When the overall results of the study are considered, it is evident that the effects of feed additives are influenced by several factors, particularly fish species and environmental conditions. Therefore, given the positive effects observed on pigmentation and growth in this study, it is recommended that further detailed investigations be conducted on leonardite, a natural, harmless and cost-effective additive, using different fish species.

## Figures and Tables

**Table 1:** Chemical composition and characteristics of leonardite ^1^.

	%		
SiO_2_	13.68	SiO_2_/Al_2_O_3_	1.93
Al_2_O_3_	7.07	BET Surface Area	12.253 m^2^/g
MgO	0.11	pH	3.15
K_2_O	0.454		
CaO	0.323		
Na_2_O	<0.014		
Fe_2_O_3_	1.238		
P_2_O_5_	0.055		
Na	0.773		
Mg	0.9927		
Al	7.32		
Si	36.65		
K	0.1969		
Ca	2.777		
Mn	0.02223		
Fe	0.9889		
P	0.0254		
Se	4.0 × 10^−5^		

^1^ Leonardite samples were provided by Kütahya Chemistry, Kütahya, Turkey.

**Table 2:** Water parameters at the end of the experiment for 90 days (Mean ± SE).

	Experimental Groups			
	Control (0%)	2%	6%	10%
Water temperature (°C)	23.5 ± 0.02	23.6 ± 0.01	23.5 ± 0.01	23.6 ± 0.02
pH	8.16 ± 0.18	8.12 ± 0.12	8.06 ± 0.12	8.05 ± 0.13
Dissolved oxygen (mg L^−1^)	6.48 ± 0.04	6.48 ± 0.02	6.50 ± 0.03	6.51 ± 0.02
NH_4_ (mg L^−1)^	0.8 ± 0.01	0.8 ± 0.03	0.7 ± 0.01	0.7 ± 0.02

**Table 3:** Growth parameters, survival and feed utilization of goldfish (*Carassius auratus*) fed with different diets for 90 days (Mean ± SE).

	Experimental Groups				
	Control (0%)	2%	6%	10%	*p*-Value
Initial weight, g	0.946 ± 0.001	0.945 ± 0.002	0.947 ± 0.002	0.948 ± 0.001	0.65
Final weight, g	1.676 ± 0.021 ^c^	2.140 ± 0.050 ^b^	2.379 ± 0.088 ^b^	2.822 ± 0.055 ^a^	0.01
Weight gain, g	0.730 ± 0.021 ^c^	1.195 ± 0.051 ^b^	1.431 ± 0.087 ^b^	1.874 ± 0.054 ^a^	0.01
SGR, %	0.635 ± 0.014 ^c^	0.907 ± 0.027 ^b^	1.021 ± 0.039 ^b^	1.212 ± 0.020 ^a^	0.02
FCR	1.273 ± 0.078 ^a^	0.998 ± 0.161 ^a^	0.975 ± 0.032 ^a^	0.681 ± 0.056 ^b^	0.01
Survival, %	76.19 ± 2.38 ^b^	78.57 ± 0.00 ^ab^	90.48 ± 2.38 ^a^	88.10 ± 4.76 ^ab^	0.01

Different superscript letters in a line indicate significant differences between groups (*p* < 0.05).

**Table 4:** Color parameter (L, a, b, C_ab_* and H_ab_°) measurements in skin of goldfish (*Carassius auratus*) fed with different diets for 90 days (Mean ± SE).

	Initial	Experimental Groups				
		Control (0%)	2%	6%	10%	*p*-Value
L*	66.53 ± 1.50	64.29 ± 1.93 ^a^	65.39 ± 1.30 ^a^	56.09 ± 1.57 ^b^	57.87 ± 1.57 ^b^	0.01
a*	150.50 ± 18.30	57.20 ± 15.3 ^a^	56.5 ± 14.8 ^a^	16.67 ± 7.55 ^b^	28.10 ± 11.40 ^ab^	0.04
b*	21.75 ± 0.89	20.49 ± 1.36 ^b^	26.47 ± 1.28 ^a^	27.35 ± 1.96 ^a^	25.17 ± 1.52 ^ab^	0.01
C_ab_*	154.50 ± 16.50	70.8 ± 13.8 ^a^	73 ± 13 ^a^	38.3 ± 6.83 ^b^	47.5 ± 10.2 ^ab^	0.04
H_ab_°	171.20 ± 10.70	113.97 ± 9.15 ^a^	114.20 ± 9.41 ^a^	85.36 ± 4.68 ^b^	94.56 ± 7.06 ^ab^	0.02

Different superscript letters in a line indicate significant differences between groups (*p* < 0.05).

## Data Availability

The original contributions presented in the study are included in the article, further inquiries can be directed to the corresponding author.
